# M^1^ündlich – S(t)imulation of competency-oriented learning in physiology

**DOI:** 10.1186/s12909-026-09661-2

**Published:** 2026-06-11

**Authors:** Lisa Eiring, Sven Benson, Joachim Fandrey, Katrin Prost-Fingerle, Sandra Winning

**Affiliations:** 1 Institute for Physiology, University of Duisburg-Essen, University Hospital Essen, Hufelandstraße 55, Essen, 45147 Germany; 2 Institute for Medical Education, Center of Translational Neuro- and Behavioral Sciences (C-TNBS), University of Duisburg-Essen, University Hospital Essen, Essen, Germany

**Keywords:** Peer teaching, Oral examination training, Communication skills

## Abstract

**Background:**

Medical students often enter oral exams with insufficient preparation, especially in articulating knowledge under stress. To improve communication and structured answering, we developed a peer-teaching-based exam training simulating oral exams.

**Methods:**

A total of approximately 190 third-semester medical students participated in the study. In groups of five, they alternated between the roles of teacher or learner. They simulated two oral exams under tutor (trained medical students of higher semesters) supervision with a time interval of three months. Each session included extensive structured feedback. This study was designed as a large-scale, curriculum-integrated, quasi-experimental study of a peer-led oral exam training intervention. The Ethics Committee of the Medical Faculty of the University of Duisburg-Essen had approved the study. Participation was voluntary and based on informed consent.

**Results:**

PT-BET was associated with increased students‘ confidence in their own competences during exams and reduced perceived exam stress. After participation students reported higher self-assessment scores, with more students rating themselves higher and fewer reporting poor performance. Stress levels decreased in the second round, with more students experiencing no or only mild stress. Most students appreciated the peer-group format being effective and high numbers planned to include it in their prospective preparation for oral exams. The tutors also noted an improvement after the second round, particularly in terms of the quality of the students’ presentations: the depth of information and the structure of the answers had improved considerably. Students’ later exam outcomes showed a favorable trend in internal follow-up exams and the oral-practical part of the first section of the German National Medical Licensing Examination (M1).

**Conclusions:**

Peer-based simulation of oral exams may support communication skills and exam awareness while being associated with reduced stress. It offers a valuable complement to the traditional medical curriculum and is easily adaptable to other disciplines.

## Background

### The importance of oral examinations and communication skills

The first section of the German National Medical Licensing Examination (M1) is taken after the first two years of study and tests knowledge in key preclinical subjects. The examination consists of written and oral-practical components. The oral-practical component tests the three major preclinical subjects of anatomy, biochemistry, and physiology. These requirements are codified in the “Approbationsordnung für Ärzte” (German medical licensing regulations): § 24 and § 30, which define the oral-practical component as a central examination format for both M1 and the third section (M3). Especially in physiology, success requires not only factual recall but also the ability to explain complex, context‑dependent processes clearly. Thus, medical knowledge alone is not enough: students must also develop their communication skills in order to convey their knowledge in a clear and structured manner. The Basel Consensus Statement of the Society for Medical Education identifies communication and social competence as key learning objectives in medical studies. These include the ability to recognize one´s own strengths and weaknesses, to take nonverbal aspects of communication into account, and to be willing to collaborate with others [1]. Their relevance is particularly evident in everyday clinical practice: effective communication by physicians has a significant impact on patients’ satisfaction and compliance, resulting in a considerable influence on health outcomes [2, 3]. Medical students are aware of this and recognize the importance of social skills [4]. However, there are currently significant shortcomings in the development of these competences [4, 5]. Current literature associates the high demands of medical studies and its high stress burden with a significantly increased prevalence of depression among students [6, 7]. This finding has also been demonstrated among German medical students [8, 9]. Oral examinations (OE) are often considered to be particularly stressful and are sometimes described as a “collective trauma” or “specter” [10, 11]. Students’ stress reactions range from exam anxiety to panic attacks and mental breakdown [10]. Important aims, such as reproducing knowledge in an exam, can no longer be achieved. This can result in a cycle of avoidance behavior, leading to exams being postponed or joint study preparations becoming less frequent. The lack of routine, in turn, increases stress levels and fear of failure [11]. Consequently, training programmes that allow safe OE practice are increasingly needed.

### Peer teaching

Peer teaching (PT) – learning through assistance from students of similar status – and its near‑peer variant (higher‑semester tutors) foster subject mastery, learning skills and a democratic learning culture [12]. A special form of this is “near-peer teaching”, in which students with more experience act as tutors to teach lower-semester students [13]. Tutorials in small groups are considered by students to be significantly more effective [14]. Small‑group PT therefore improves knowledge transfer, confidence, social belonging [12, 15, 16] and, for tutors, deepens their own expertise [17]. The proximity between tutors and participants creates a trusting learning atmosphere, makes it easier to disclose gaps in knowledge, and promotes constructive mutual feedback [18]. The latter is a key way of providing targeted support for students’ development [19] and is important from early on [20]. In addition, studies show that participants of PT achieve better exam results [15].

### Didactic triangle and didactic circle

Wildt´s didactic triangle model provides a suitable framework for the theoretical classification of the PT format. It describes the interdependence of teachers, learners and knowledge [21]. Teaching promotes learning processes and requires adopting the perspective of learners. As early as 1968, the “Theses on University Didactics” [22] emphasized the “exploration of students’ needs and motivations” as a central concept of modern university didactics [23].

Students take over both the role of teachers and learners during the herein described exam training. In the teaching role, students examine the structure of the learner´s answers and provide guidance; in the learning role, they reflect on and deepen their own knowledge. This is particularly important in physiology, where complex processes require structured explanation and precise communication. Thus, the didactic triangle is reflected not only theoretically but also in practice.

Complementary, the didactic circle describes the flexibility of teaching and learning [24]. It includes methods, content, social forms, and evaluations, which can be adapted depending on emphasis. In addition, the teaching-learning process is shaped by institutional, disciplinary, and societal requirements [21]. These theoretical considerations form the framework for the development and evaluation of the PT described below.

### Objective

During the COVID‑19 pandemic, a national survey highlighted students’ need for targeted OE preparation [25]. In order to conduct a targeted needs analysis, local data was analyzed to determine whether this perceived need was also evident at our institution, which revealed a decrease of approximately 20% points in students taking part in the M1 during the period of digital teaching. Thus, many students postponed the exam due to a decreased self-assessment of their competences in order to prepare more intensively and / or to catch up on learning deficits.

We therefore developed “M¹ündlich”, a peer‑tutorial for physiology aimed at (i) reducing anxiety, stress and “blackouts”, (ii) improving exam rhetoric and structuring, and (iii) encouraging small‑group preparation. The study examined whether PT‑BET increases self‑assessment, confidence and peer‑group use, lowers stress, improves tutor ratings, and affects oral M1 grades.

## Methods

### Study design and study population

This is a prospective, monocentric large-scale, curriculum-integrated, quasi-experimental study of a peer-led oral exam training intervention conducted at the Institute of Physiology at the University of Duisburg-Essen. Eligible participants were third-semester medical students who were at least 18 years of age and gave their written consent to participate. A total of 189 and 178 students of 212 enrolled in the semester participated in the two training rounds, respectively, and 188 students participated in the midterm exam. Participation was voluntary and without risk to the participants. A control group within the same semester was not included, as it did not seem reasonable to withhold exam training from a randomly selected group.

### Exam training procedure

The PT-BET was offered in parallel with the physiology lecture in the third semester and comprised two training rounds, referred to as T1 and T2. T1 took place from November 18 to November 22, 2024, and T2 from January 16 to January 21, 2025.

**Fig. 1:**
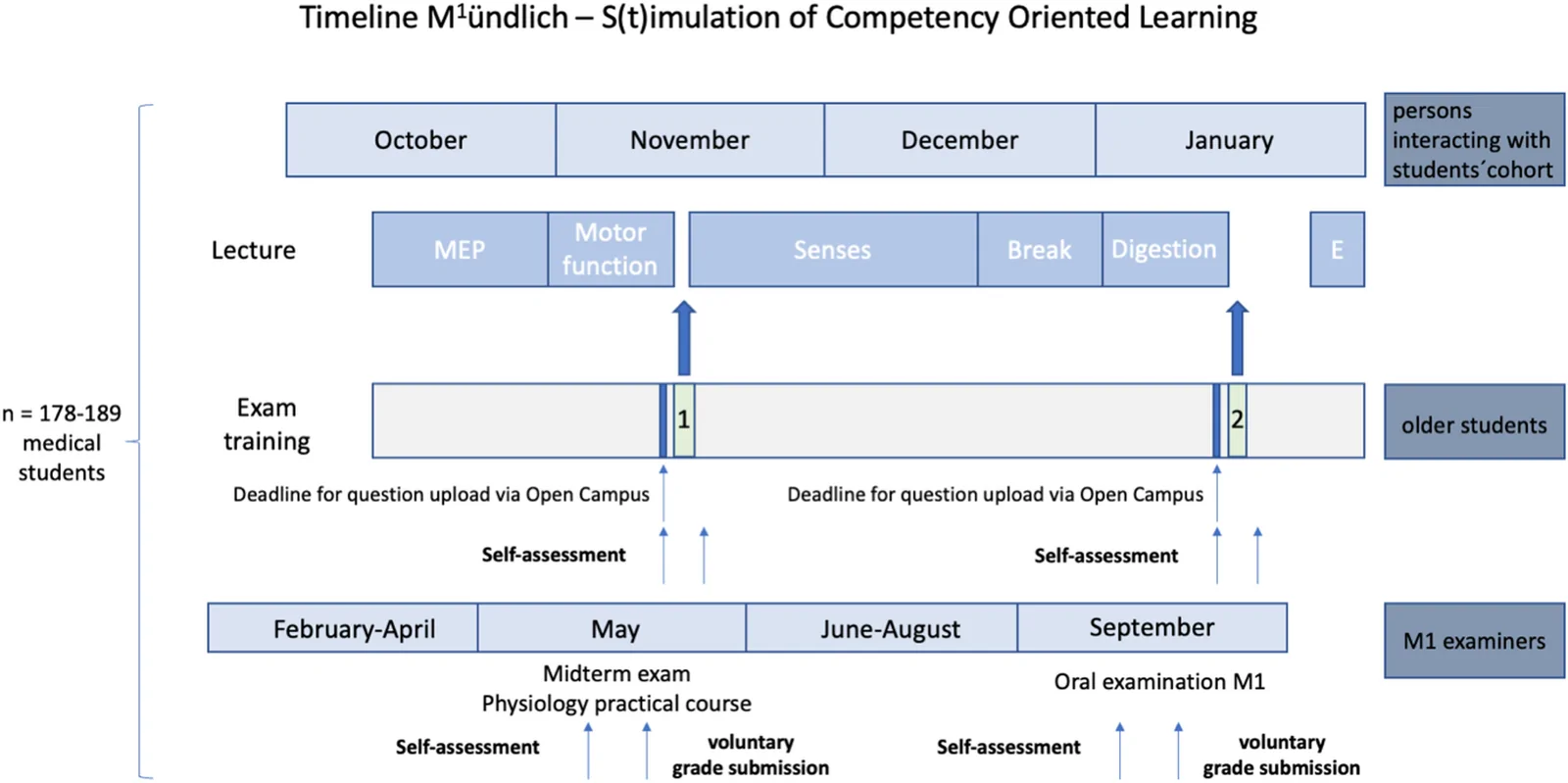
Timeline of the peer-teaching-based exam training “M1ündlich - Simulation of Competency Oriented Learning, conducted in 2024–2025. The figure shows the two training rounds in the context of the physiology lecture and the respective times of the self-assessments. In addition, the midterm exam and the oral part of the first section of the medical examination (M1) are shown in chronological order

**Fig. 2:**
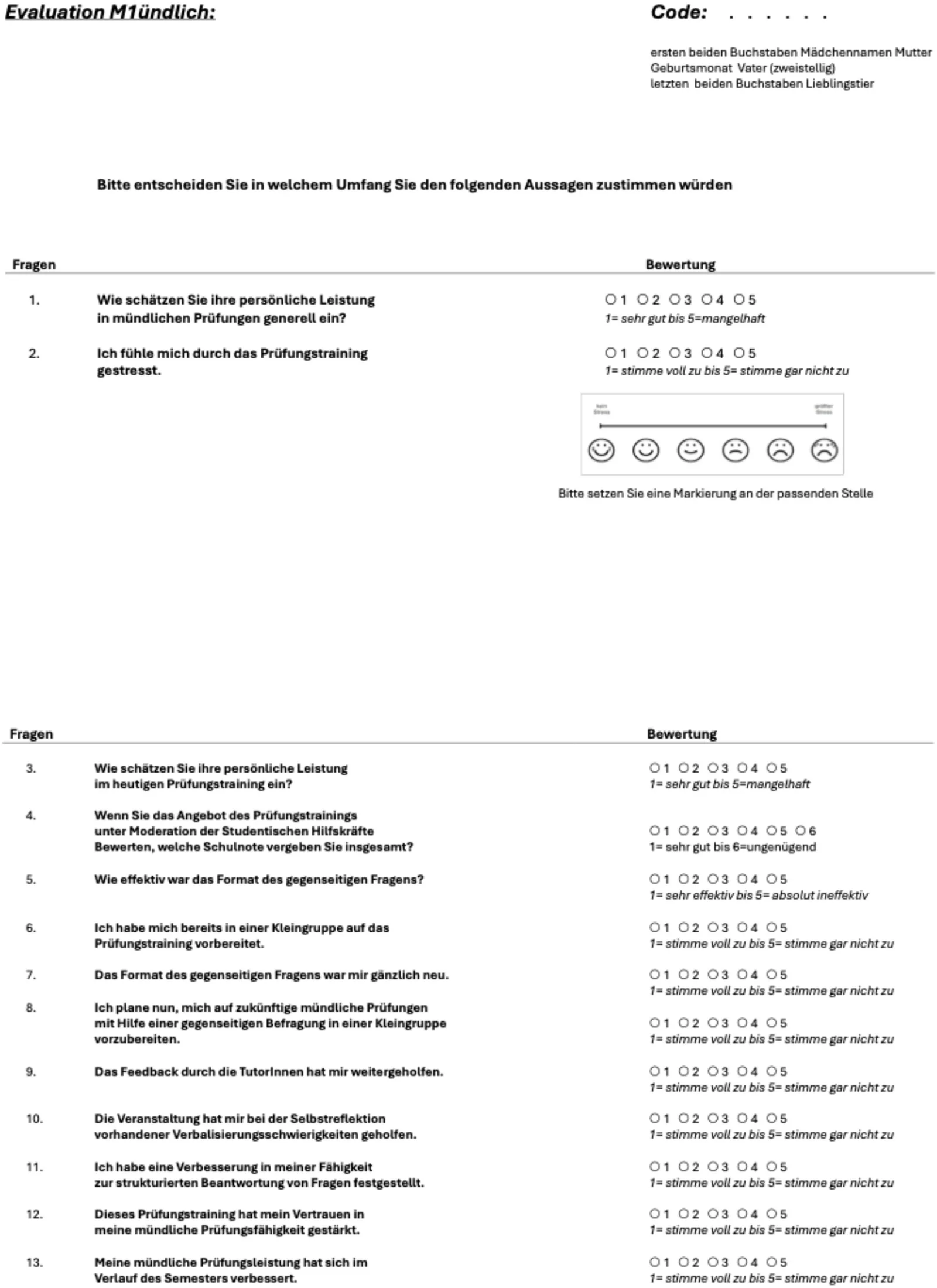
Evaluation form used in the study. The front side contained pre-training items for self-assessment of oral exam performance (Likert scale) and for recording perceived stress levels (Perkhofer Stress Scale). The back included post-training items for evaluating the exam training and perceived improvement in oral exam skills, collected using a Likert scale

The teaching content of the training sessions was thematically adapted to the respective lecture content (see Fig. 1). The students worked on previously submitted student-generated questions in groups of five under the guidance of trained tutors. The tutors completed a structured, two-day preparatory training session led by PD Dr. rer. Nat. Sandra Winning and Dr. rer. nat. Katrin Prost-Fingerle (holding a MQ1 certificate, certified by the MedizinDidaktikNetz (MDN) Germany). The training included standardized instructions on conducting sessions, providing feedback and applying predefined assessment criteria. It addressed typical challenges in oral exams, common difficulties faced by students, and strategies for answering questions effectively. In addition, case-based examples were used to calibrate the tutors’ assessments and ensure consistency in the evaluation and training procedures. Within the group, the students alternated between the roles of teacher and learner by asking each other questions randomly drawn from a pre-selected question pool. The aim of the exam training was to practice the structure, expression, and content depth of OE and to gain confidence in dealing with exam situations. After each round, the students received detailed feedback from the tutors, which was based on a standardized evaluation form and covered the categories of structure and density of answers, presentation quality, expression and precision. The evaluations (see Fig. 2) were based on a six-point scale from 1 (very good) to 6 (unsatisfactory).

### Data collection

A standardized evaluation form was used in both rounds of exam training and in the midterm exam to record subjective perceptions. At the beginning, students generated an individual identification code to anonymously assign the data between the survey dates [26]. The evaluation form was completed by the students in two phases: The front page was completed immediately before the start of the training and included a self-assessment of their own performance in OE using a five-point Likert scale [27], as well as their subjectively perceived stress level, which was assessed using the Perkhofer Stress Scale, a six-level faces-based rating scale [28].

The back side was completed immediately after the training and recorded the students ‘perceptions of the exam training, including their self-assessment of their own performance, their evaluation of the tutors ‘performance, and their perceived effectiveness of the mutual questioning format. In addition, previous experiences with and future plans for small group preparation, the novelty of the training format, and the perceived usefulness of tutor-feedback were also collected. Other aspects recorded included self-reflection on verbalization difficulties, perceived improvements in structured responses to oral exam questions, confidence in one´s own oral exam skills, and perceived changes in oral exam performance over the course of the semester. All parameters were assessed using a five-point Likert scale.

After completion of the oral part of the M1 OE (“1. Abschnitt der ärztlichen Prüfung in Deutschland” according to the “Approbationsordnung für Ärzte vom 27. Juni 2002 (BGBl. I S. 2405), a digital follow-up survey of the students was conducted subsequent to their M1 OE.

To the best of our knowledge there are no published questionnaires fitting our PT-BET. We therefore established an individual evaluation concept in close collaboration with the Institute of Medical Education, which was reviewed by the local Ethics committee prior to study approval. The questionnaires were applied in an unchanged form across all measurement time points, ensuring internal consistency of the instrument.

### Data protection

All data was collected pseudonymously and stored on password-protected computers of the Institute of Physiology. It was not possible to trace the data back to individual persons. The study was approved by the Ethics Committee of the Medical Faculty of the University of Duisburg-Essen (24-12071-BO).

### Statistical analysis

The data was analyzed using GraphPad Prism statistical software (version 8.4.3 for Windows, GraphPad Software, Boston, Massachusetts, USA, www.graphpad.com). Descriptive parameters (median, frequencies, percentage distributions) were calculated. The objective of the study was to evaluate the effects of the PT-BET on student´s perceptions of OE situations, including subjective stress levels, self-assessment, confidence in exam performance, preparation in small groups and student´s intention to prepare for exams in peer groups. To assess the ordinal association between the students’ perceived stress levels, their trust strengthening and the preparation for oral trainings in small groups with their individual self-assessment we calculated the Kendall rank coefficient (Kendall´s tau: -1: perfect negative correlation, =: no correlation, + 1: perfect positive correlation) and the respective p-values with Numiqo (https://numiqo.de/). In addition, the grade distributions in the midterm exam (years 2023–2025) and in the oral part of M1 (2018 as pre-pandemic reference point, 2021, 2024 and 2025) were analyzed using comparative data from previous years. For each year, the frequency of each grade was recorded and analyzed separately. A significance level of *p* < 0.05 was considered statistically significant for all analyses.

## Results

### Self-assessment

**Fig. 3:**
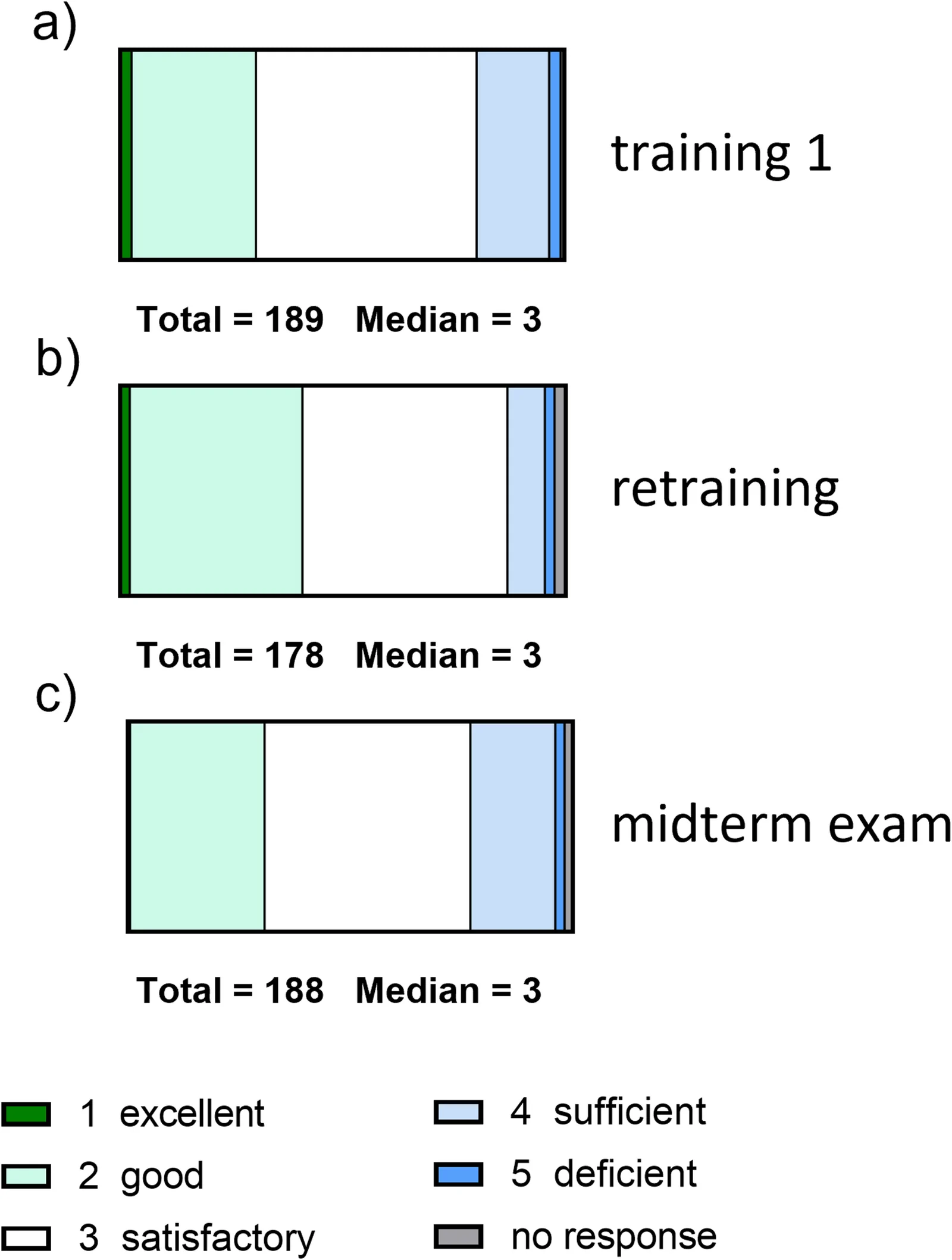
Students’ self-assessment before the two training rounds and the midterm exam. The figure shows the distribution of ratings on a five-point Likert scale (1 = excellent, 5 = deficient) at the three measurement points (**a**) training, (**b**) retraining, (**c**) midterm exam

The students’ self-assessment in OE was recorded at three time points: prior to training 1 (T1) and retraining (T2) and before the midterm exam (MTE) (see Figs. 3a-c). In T1 (*n* = 189), the median was 3. Almost half of the students (49.7%) rated themselves in the middle range, while only around 3% gave themselves a self-assessment of 1 (excellent), and another 3% of 5 (deficient). In T2 (*n* = 178), the median remained at 3, but the distribution slightly shifted towards more favorable assessments. The proportion in grade 2 increased by 10.8%, while grade 4 decreased by 8%. In the MTE (*n* = 188), the median shifted to 4. Around half (46.3%) of the students ranked themselves at grade 3 as before, while the proportion in grade 4 expanded to 19.1%.

### Stress level

**Fig. 4:**
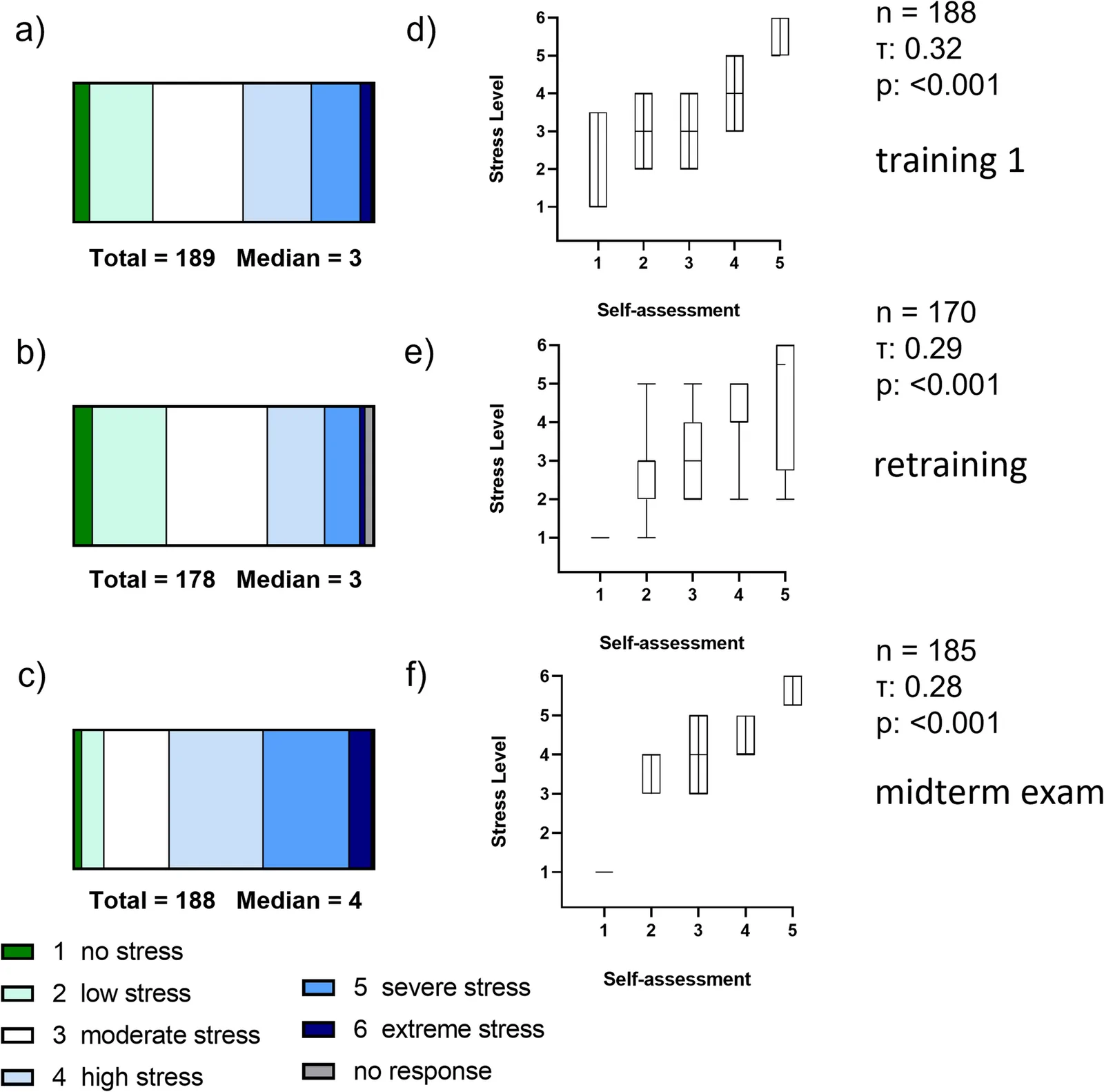
Students’ stress levels before the two training rounds and the midterm exam, and their correlation with self-assessment. Bar charts (left) show the distribution of self-reported stress levels, measured on a six-point Likert scale (1 = no stress, 6 = extreme stress) at the three measurement points (**a**) training, (**b**) retraining, (**c**) midterm exam. Boxplots show how individual students classified their self-assessment and stress levels at the same time points (**d**) training, (**e**) retraining and (**f**) midterm exam. To evaluate the individual, ordinal association between self-assessment and stress level, Kendall´s τ has been calculated as indicated

The students’ stress levels were recorded simultaneously with self-assessment (see Fig. 4a, c and e). In T1 (*n* = 189), the median was 3. A high proportion of students (43.3%) reported a high stress level (category 4–6), while 29.6% of participants classified themselves in category 3 and a total of 26.5% reported a rather low stress level (categories 1 and 2) (see Fig. 4a). In T2 (*n* = 178), the median remained at 3. The proportion of low stress levels (categories 1 and 2) increased by 4.4%, reaching 30.9%, while the proportion of high stress levels (categories 4–6) decreased by 10.7%, reaching 30.9% (see Fig. 4b). At the time of the MTE (*n* = 188), the median remained at 3, but the distribution shifted significantly towards higher stress levels again. The largest proportion of students, 67.5%, now classified themselves in categories 4–6, while only 10.1% of participants reported low stress levels (categories 1 and 2) (see Fig. 4c). There was a positive (Kendall´s τ = 0.28–0.31) and significant correlation between stress levels and self-assessment at all three time points. Students who rated their exam performance as poorer, accordingly reported higher stress levels (T1: *p* < 0.001; T2 *p* < 0.001; MTE: *p* < 0.001) (see Figs. 4d-f).

### Trust strengthening

**Fig. 5:**
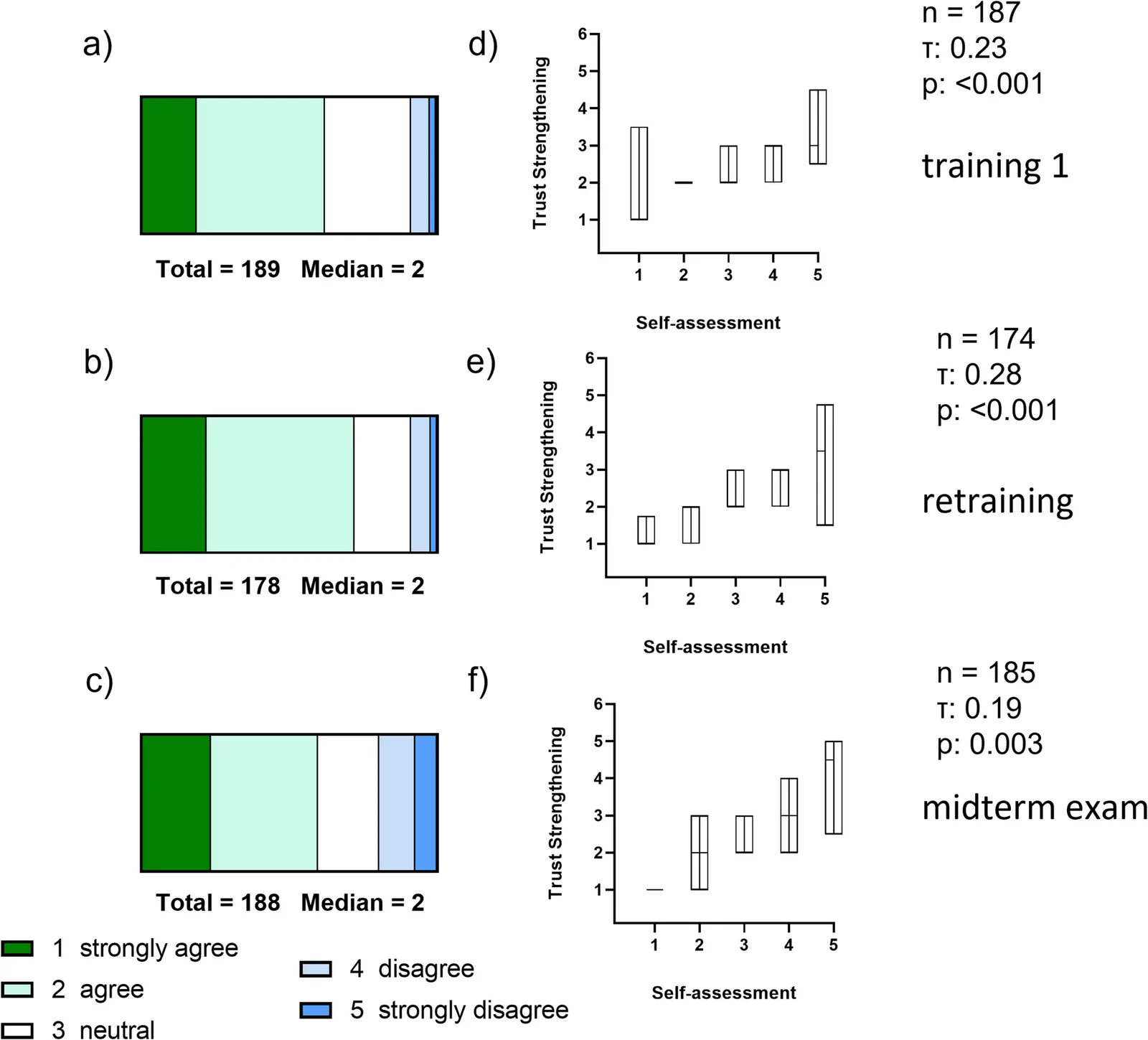
Development of student’s confidence in their own performance over the three measurement points and their correlation with self-assessment. The bar charts (left) show the distribution of student´s perceived confidence in their own performance, rated on a five-point Likert-scale (1 = strongly agree, 5 = strongly disagree), after the two training rounds (**a**, **b**) and after the midterm exam (**c**). Boxplots (right) show how individual students classified their self-assessment and trust strengthening at the same time points (**d**) training, (**e**) retraining and (**f**) midterm exam. To evaluate the individual, ordinal association between self-assessment and trust strengthening, Kendall´s τ has been calculated as indicated

The students’ perception of an increased confidence due to participation in the training remained at a high level across all three measurement points (see Figs. 5a-c). The median was 2 in both T1 and T2 and in the MTE. A total of 61.9% of students in T1 (*n* = 189) stated that participating in the PT-BET had strengthened their confidence in their own abilities (categories 1 and 2) (see Fig. 5a). In T2 (*n* = 178), this proportion increased by 10% to 71.9%, while neutral assessments (category 3) decreased accordingly (see Fig. 5b). In the MTE (*n* = 188), the proportion of affirmative assessments (category 1 and 2) remained predominantly high, but showed a slight increase in negative assessments to 19.6% (categories 4 and 5) (see Fig. 5c). There was a significant positive correlation between the students’ self-assessment and their perceived confidence strengthening at all three time points (T1 and T2: *p* < 0.001; MTE *p* = 0.003; Kendall´s τ = 0.19–0.28), with students who rated themselves higher also reported a greater increase in confidence (see Figs. 5d-f).

### Peer-group preparation

**Fig. 6:**
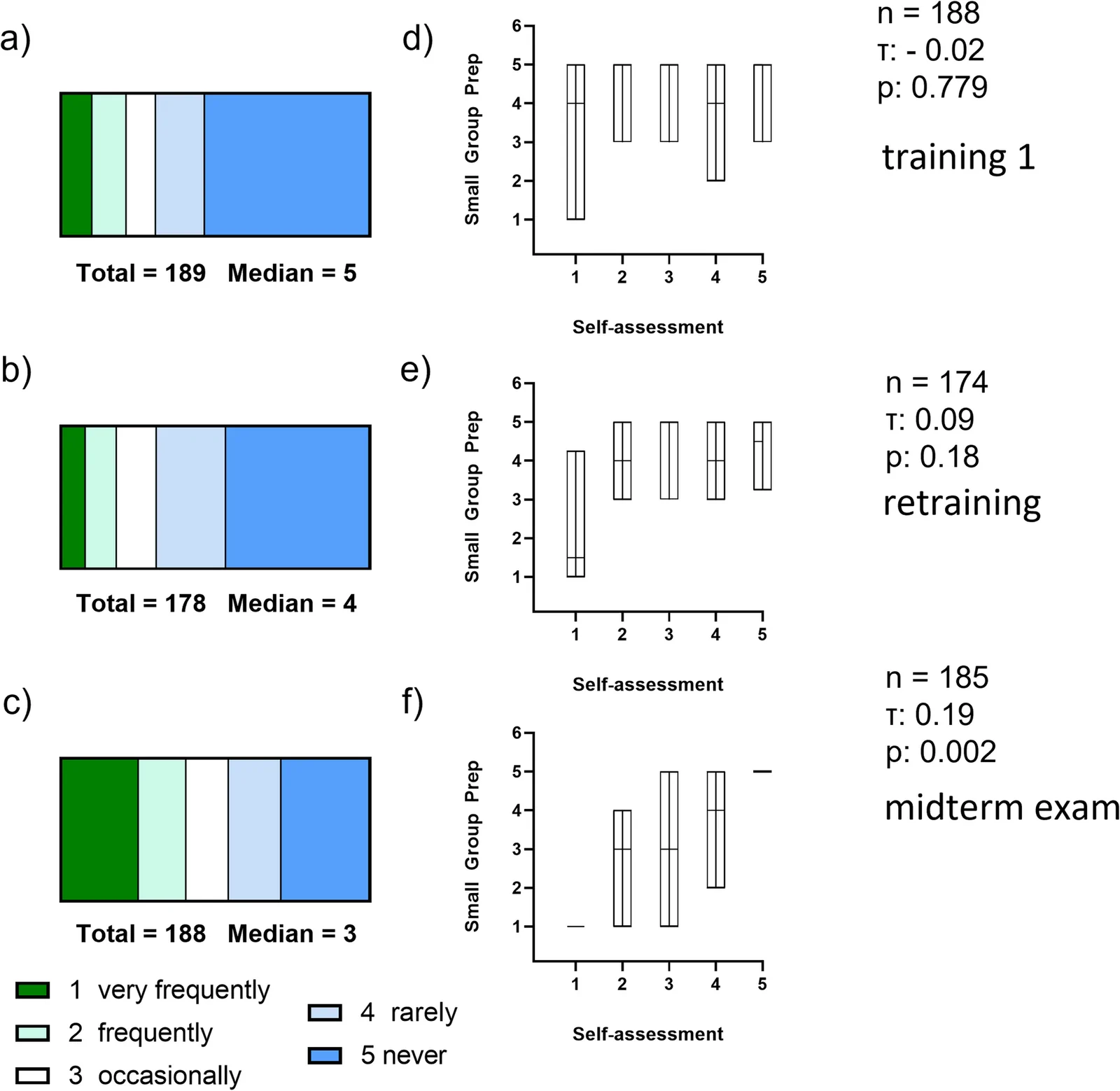
Use of peer group preparation before the two training rounds and the midterm exam, and its correlation with self-assessment of exam performance. The bar charts (left) show how often students reported using small group preparation, rated on a five-point Likert scale (1 = very often, 5 = never), at the three measurement points (**a**) training, (**b**) retraining, (**c**) midterm exam. Boxplots (right) show how individual students classified their self-assessment and peer group preparation at the same time points (**d**) training, (**e**) retraining and (**f**) midterm exam. To evaluate the individual, ordinal association between self-assessment and peer group preparation, Kendall´s τ has been calculated as indicated

The use of peer groups for the students ‘preparation was recorded at all three survey points (see Figs. 6a-c). In T1 (n = 189), the median was 5. Significantly more than half of the students (69.3%) stated that they rarely or never prepared themselves for an (oral) exam in a peer group (categories 4 and 5), while only around 20% of students reported frequent to regular use (categories 1 and 2) (see Fig. 6a). In T2 (n = 178), the median reduced to 4 and there was a slight increase from category 5 to 4. The proportion of students who rarely prepared in a peer group was 22.5%, while the proportion of students who had never prepared in a peer group remained high at 46.6% (see Fig. 6b). At the time of the MTE (n = 188), exam preparation in a peer group was used to a significantly higher extends. The median decreased to 3. A quarter of the students (25%) now reported a regular preparation in a peer group (category 1), while only 28.7% of the students stated that they had not used any peer group preparation at all (see Fig. 6c). No correlation between self-assessment and peer group preparation could be demonstrated in either training round (T1: p = 0.86; T2: p = 0.10) (see Fig. 6d and e). In contrast, the MTE showed a significantly positive correlation (p = 0.0011), meaning that students who prepared in peer groups had a better self-assessment while they were directly facing the midterm exams (see Fig. 6f).

### Peer-group concept

**Fig. 7:**
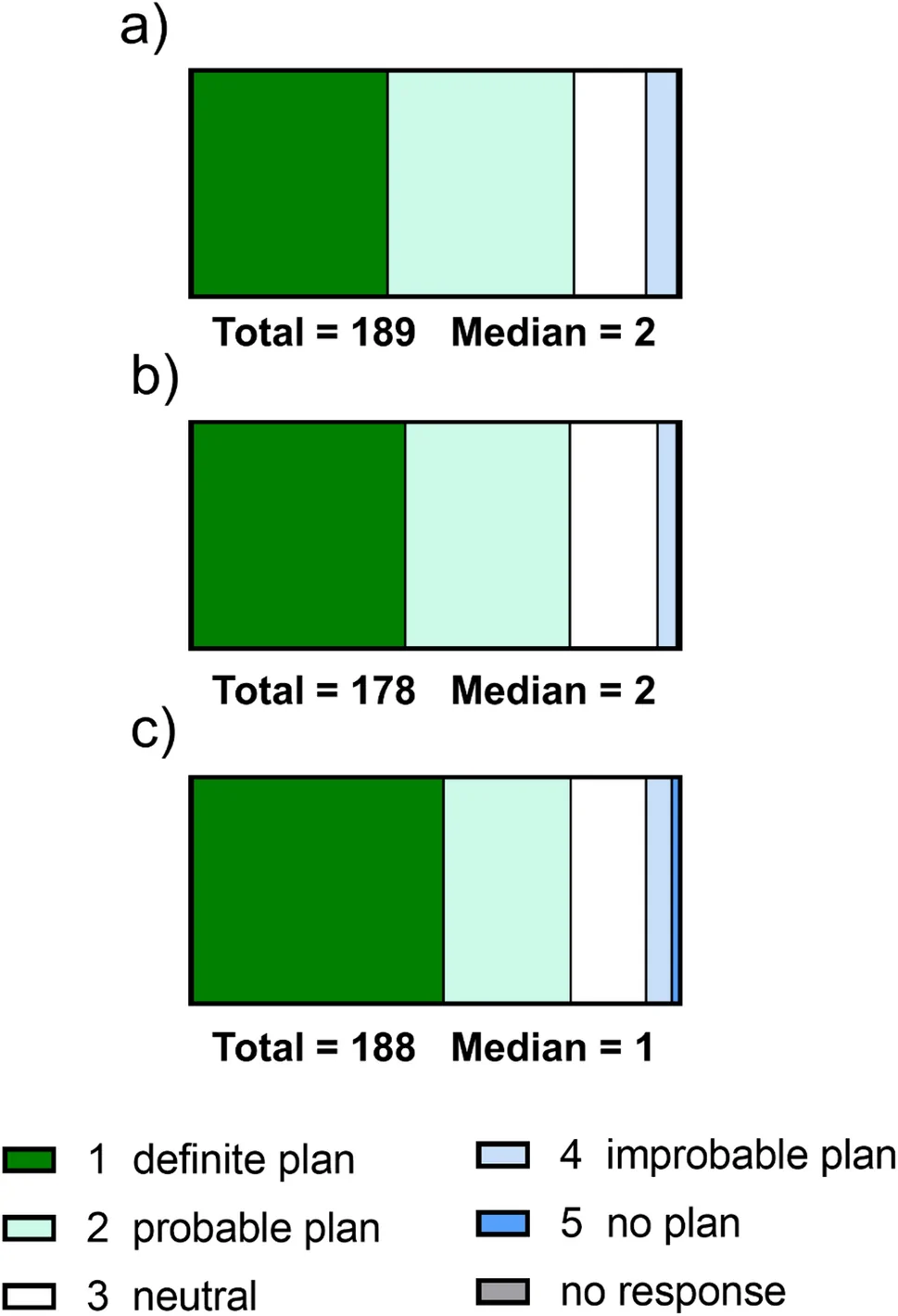
Students’ intention to prepare for exams in peer groups after the training and the midterm exam. The figure shows the distribution of response on a five-point Likert scale (1 = very likely, 5 = not at all likely) at the three measurement points: (**a**) training, (**b**) retraining, and (**c**) midterm exam

The planning to prepare themselves in peer groups for following exams was recorded at three time points (see Figs. 7a-c). In T1 (*n* = 189), the median was 2. The majority of students (78.3%) stated that they wanted to prepare for future exam formats in a peer group (category 1), while only 6.8% of students denied this (category 5). In T2 (*n* = 178), the median remained at 2. A total of 77.5% of students continued to favor peer groups for exam preparation, while the proportion of students who did not intend it decreased to 4.5%. In the MTE (*n* = 188), the median shifted to 1, meaning that the proportion of strongest agreement (1) increase by 7.7% to 51.6%.

### Individual changes between T1 and T2

**Fig. 8:**
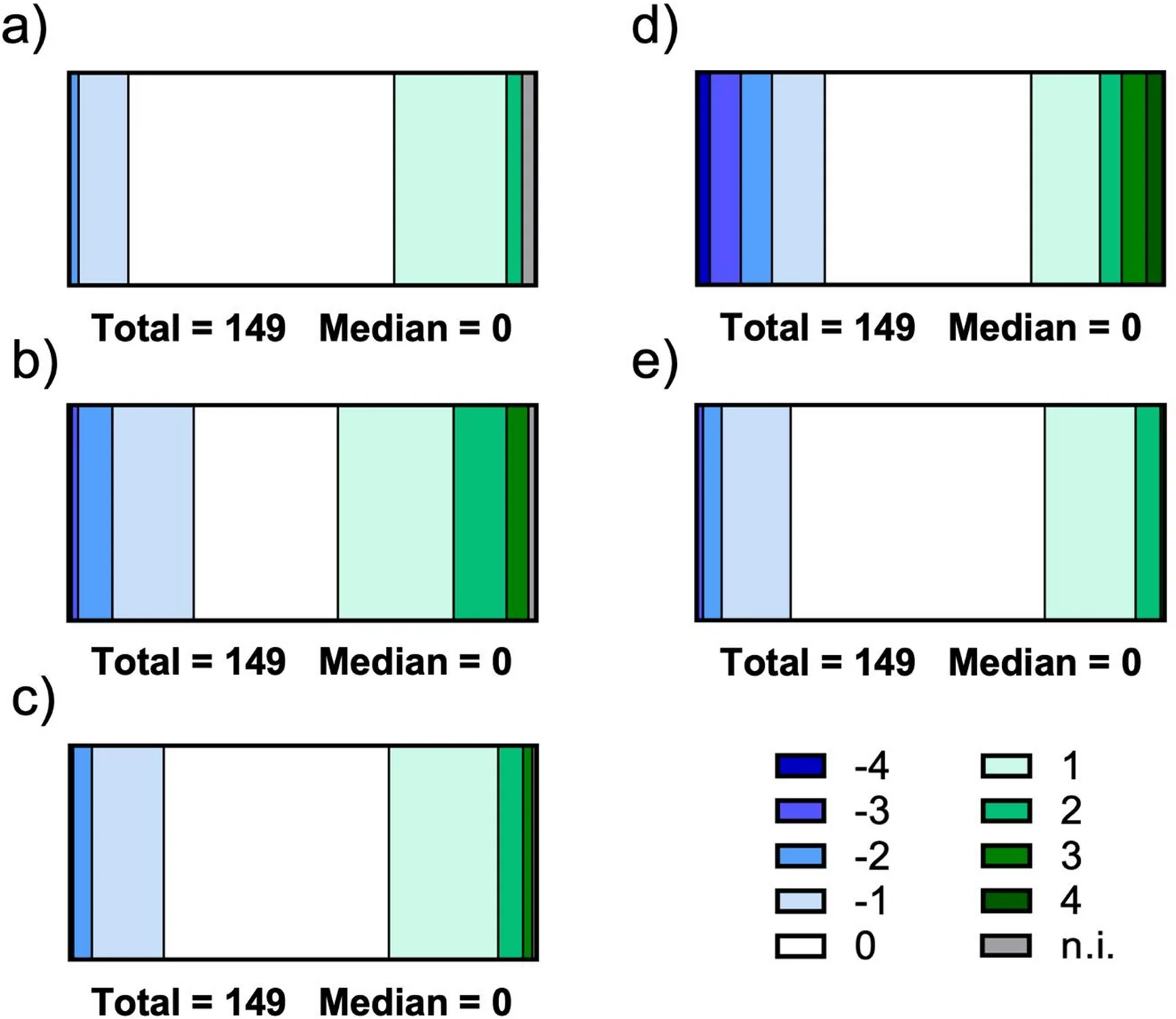
Individually perceived changes in students’ responses between the first and second training rounds across the five assessment categories. The figure shows the distribution of paired response changes based on anonymous participant codes for (**a**) self-assessment, (**b**) stress level, (**c**) confidence strengthening, (**d**) peer-group preparation, and (**e**) students’ intention to prepare for exams in peer groups. Changes were categorized as unchanged (white), worsened (blue: -4 to -1), or improved (green: 1 to 4)

Based on anonymized codes, individual changes in students’ responses between T1 and T2 were analyzed for self-assessment, stress level, trust strengthening, peer-group preparation and planning of peer-group preparation (*n* = 149 each) (see Fig. 8a-e). For all assessed categories, the median did not change between T1 and T2 (Figs. 3, 4, 5, 6 and 7a/b).

For self-assessment, 57% of students showed no change between T1 and T2, while 27.5% reported an improvement and 12.8% a decrease. Overall, 2.7% of cases had missing values (see Fig. 8a). Interestingly, a more detailed analysis of self-assessment changes showed that the majority of improvements occurred among students with initially low self-assessment scores (categories 4 and 5) (data not shown).

With regard to stress levels, 30.9% of students showed no change over time. For 40.9% of students, stress levels improved, while they worsened for 26.8%. Missing values accounted for 1.3% (see Fig. 8b). Again, students with initially high stress levels (categories 4–6) showed the greatest improvements (data not shown).

For trust strengthening, 48.3% of students showed no change between T1 and T2. An increase was reported by 29.5%, while 20.1% showed a decrease. Missing data were present in 0.7% of cases (see Fig. 8c).

44.3% of students of the students showed no changes in their preparation for the training sessions. They did not use peer-group preparation, neither in the first nor in the second round. 28.2% reported to have used more peer-group preparation in round 2, whereas 27.5% reported a decrease (see Fig. 8d).

Analysis of the students’ planning of peer-group preparation for further exams showed that 54.5% of students did not change their opinion between T1 and T2. A total of 25.5% of students planned to integrate more frequent peer-group preparation, while 20.1% planned to use it less (see Fig. 8e).

### Assessment by tutors

**Fig. 9:**
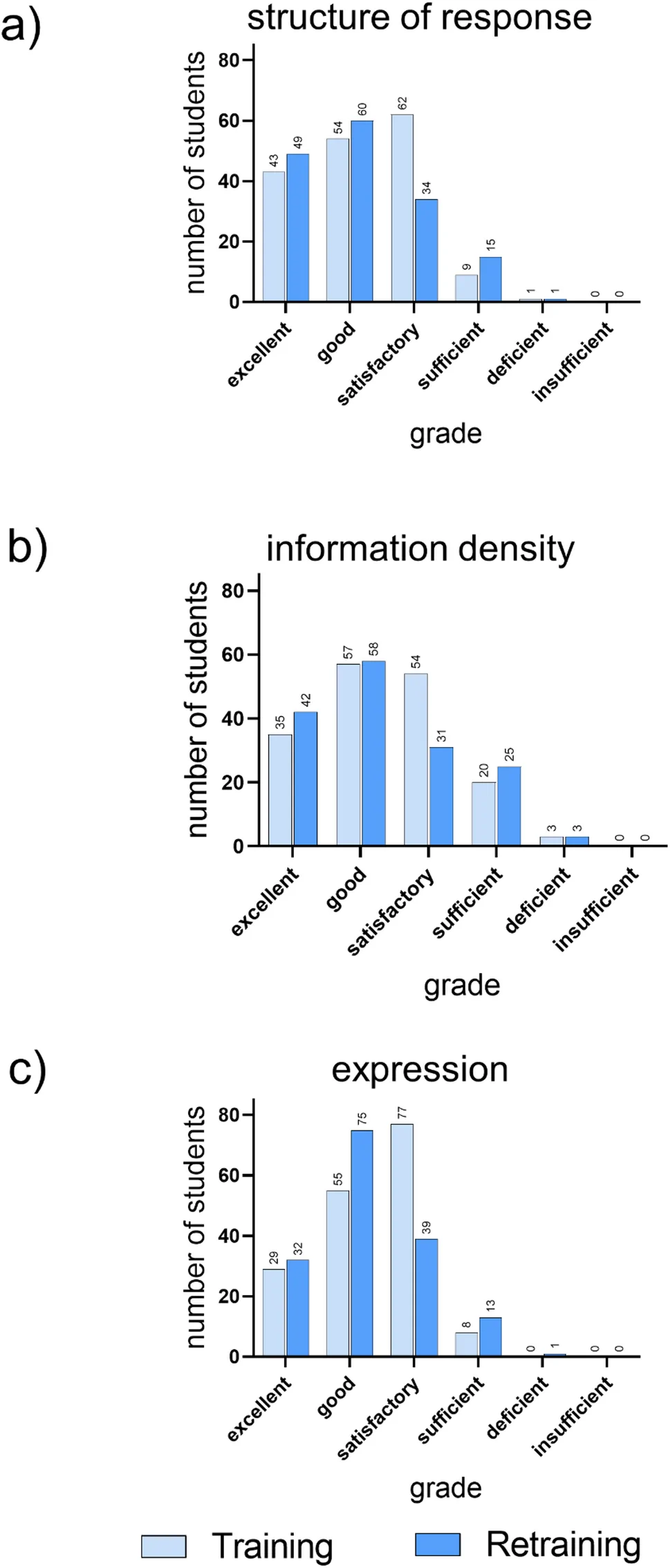
Comparison of tutor evaluations between the first and second rounds of exam training. The bar charts show the distribution of grades awarded (1 = excellent, 6 = deficient) in the categories (**a**) structure of response, (**b**) information density and (**c**) expression

In order to objectively assess the presentation performances, the tutors carried out a standardized evaluation in both training rounds (suppl. Figure 9). In terms of information density, there was a significant improvement between T1 and T2 (see Fig. 9a). While 53.80% of the students in T1 (*n* = 171) were rated in the best categories 1 and 2, this parameter increases to 62.50% in T2 (*n* = 160). Correspondingly, the number of average and weaker ratings (3–5) decreased. The structure of the given answers improved (see Fig. 9b). In T1 (*n* = 171), 56.73% of the ratings fell into categories 1 and 2, whereas in T2 (*n* = 160), this proportion of ratings in categories 1 and 2 increased by 17.76%. The proportion of average ratings decreased from 45% to 8%. The distribution thus shifted significantly in favor of better ratings. All three rating criteria showed a consistent increase in performance over the course of training (see Figs. 9a-c).

### Grade distribution in the midterm exam

**Fig. 10:**
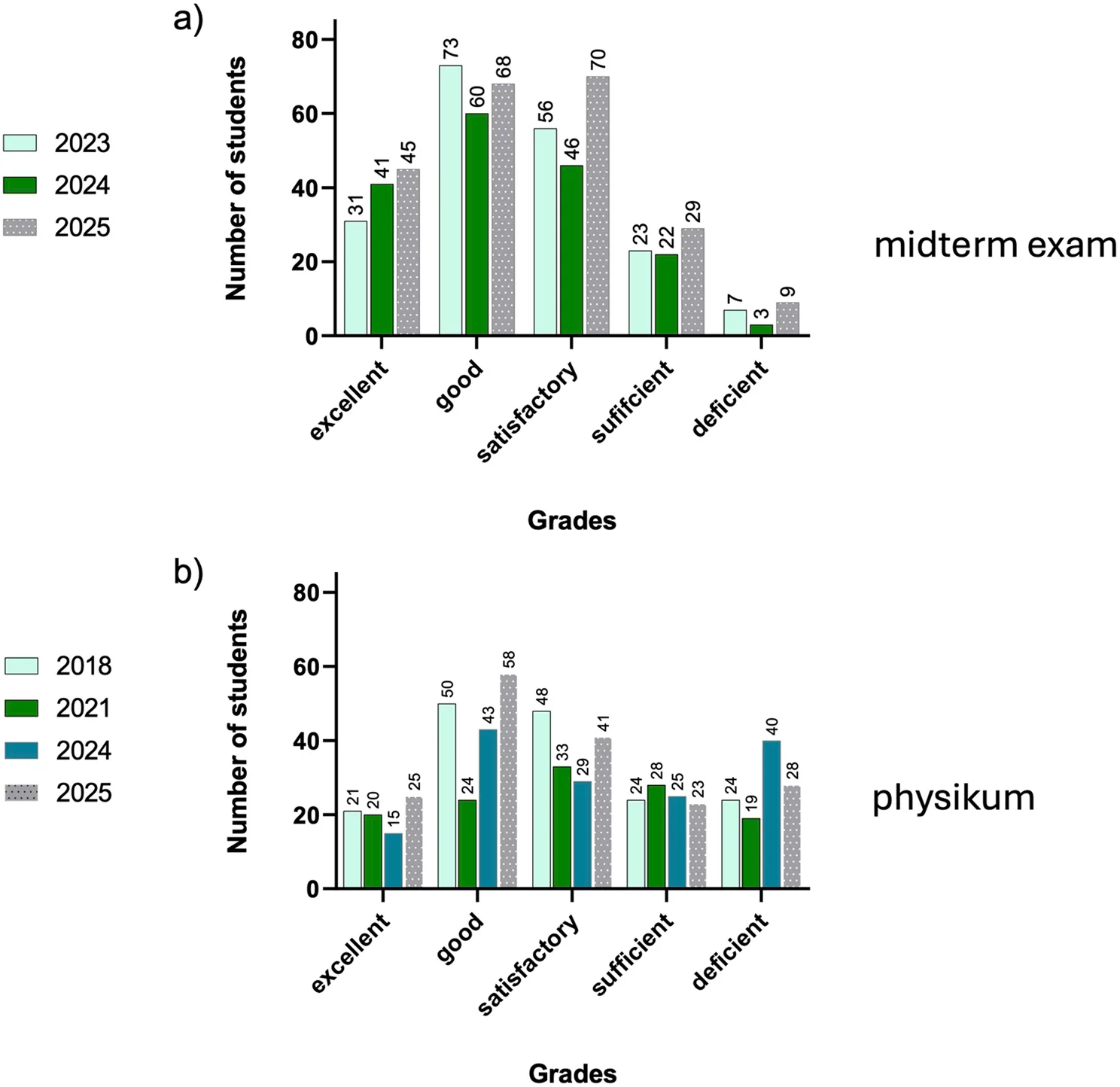
Grade distribution in the midterm exam (**a**) and in the oral state examination (**b**) before and after the introduction of peer-teaching-based exam training. Subfigure (**a**) shows the distribution of grades (1 = excellent, 6 = deficient) in the midterm exam for the years 2023, 2024 and 2025. Subfigure (**b**) shows the corresponding grade distribution in the oral state examination for the years 2018, 2021, 2024 and 2025

The distribution of grades in in the MTE (administered by university lecturers) was recorded for the years 2023 to 2025 (see Fig. 10a). In 2023 (*n* = 190), most students received a grade of 2 or 3 (67.89%), while the proportions of the best (1) and worst grades (5) were comparatively low at a total of 20.01%. In 2024 (*n* = 172), the distribution of grades shifted slightly in favor of better performance: the proportion of the best grade (1) increased by 7.52%, while the proportion of the worst grade (5) declined. In 2025 (*n* = 221), there was an almost equal distribution between grades 2 (30.77%) and 3 (31.67%), while the proportion of the worst grade (5) increased slightly. Overall, the majority of students in all three years performed generally positive.

### Grade distribution in the M1 oral examination

The distribution of grades in the oral part of the M1 was examined for the years 2018 (pre-pandemic reference), 2021 (digital learning), 2024 (post-pandemic) and 2025 (PT-BET) (see Fig. 10b). In fall 2018 (*n* = 167), the largest proportion of students received grades 2 (29.9%) and 3 (28.7%), while 14.4% did not pass the exam (grade 5). In fall 2021 (*n* = 124), there was an increase in poorer performance: 22.6% of students received a grade of 4, and 15.3% did not pass the exam (grade 5). In 2024 (*n* = 152), the distribution shifted further towards poorer results. 9.9% of students achieved the top grade of 1, while around a quarter (26.3%) did not pass the exam (5), which is the highest value in the comparison period. In fall 2025 in turn (*n* = 175), there was a significant improvement: 14.3% of participants received the highest grade, and the proportion of lower grades decreased again, to 13.0% for grade 4 and 16.0% for grade 5.

## Discussion

The German National Medical Licensing Exam (M1) poses a challenge for students, especially in physiology, where they have to present complex content in a structured and differentiated manner within the oral exam. Even more, accurate communication is a key competence for later clinical studies and practicing medicine. Experienced stress in oral exams is often described as a major limitation in performance [29, 30]. Therefore, we set up a peer teaching-based exam training program (PT-BET) to support medical students improve their communication competences, which may be associated with reduced perceived stress levels. This may contribute to improved personal self-assessment before and increased confidence in their oral exam abilities after PT-BET, which might be extendable to future exam or communicatively challenging situations.

PT has been shown to be associated with both subject-specific knowledge and individual learning skills in various studies and different other disciplines. Kleinert et al. found that participation in student tutorials during the introductory phase of biology correlated positively not only with exam results, but students’ perception of competence, autonomy, and social integration. Students who attended more tutoring sessions achieved greater increase in those competences than students with lower participation [15]. Similarly, a meta-analysis by Hidayat & Mohd Saad in STEM subjects (science, technology, engineering and mathematics) also showed that PT significantly improved academic performance and had smaller but still positive effects on self-concept, motivation and critical thinking, with particularly strong benefits in biology and engineering [31]. These findings are consistent with Boud, who emphasized that peer teaching not only supported academic achievement but also promoted students’ self-confidence, autonomy, and teamwork skills [16]. Topping & Ehly further argued that peer learning could foster positive social interactions, empathy, motivation and self-confidence while also strengthening tutors sense of responsibility and engagement in the learning process [12]. A systematic meta-analysis focused on the outcomes for knowledge and skills of medical students taught by peers in comparison to faculty teaching did not find significantly different outcomes [32]. Nonetheless, the authors clearly came to the conclusion that PT should be supported as it may contribute to the development of knowledge and teaching competences of the tutors in addition to those of the taught students. Building on established PT guidelines, our PT-BET exam training implemented several key strategies, including structured content planning, short active learning sessions, tutor training, systematic feedback, repeated improvement cycles, and regularly offering the session during medical studies [33].

Our data suggest that PT-BET may be associated with increased students‘ confidence in their own competences during exams while potentially being associated with reduced perceived exam stress (see Figs. 2 and 3). It should be noted that this only reflects a subjective self-assessment; no conclusions can be drawn about actual exam performance, as previous oral exam grades were not recorded and there are no oral exams in physiology before the PT-BET.

The correlations between perceived competence and stress (Fig. 4d-f) seem to highlight the importance of self-efficacy for coping with stress during exam situations. This correlation may be explained by Banduras theoretical considerations: those who felt competent assessed challenging situations as manageable and reacted with less physiological and emotional tension [34]. Studies examining self-efficacy and stress responses during OE [30], its moderating role in test anxiety [35], and the relationship with academic performance [36] have all confirmed this correlation. Previous research has shown that so-called high-stakes exams, i.e., exams that are perceived as very important, are generally associated with more pronounced test anxiety than low-stakes exams, especially when students attribute high importance to the task [37, 38]. A similar pattern was observed in our study: the midterm exam conducted by physiology faculty, which is a mandatory performance assessment and therefore a high-stakes situation, was associated with higher stress levels than the low-stakes PT-BET (Fig. 3). This finding suggests that while PT may be associated with reduced exam anxiety, frequent exposure to regular authentic examination settings may also be necessary to foster adaptive stress regulation over time.

Beyond cognitive and motivational aspects, the peer teaching format may be interpreted within the didactic triangle as an interaction between teachers, learners, and knowledge, enacted through role switching in PT-BET. This interaction occurs within learners’ individual preconditions, such as prior experiences with academic communication. Therefore, the structured peer learning environment may be particularly beneficial for students from non-academic family backgrounds by providing low-threshold opportunities to practice academic discourse and build communicative confidence.

The PT-BET suggests an increase in students’ confidence in their own competences (Fig. 4). The correlation between increased confidence and self-assessment was particularly evident: students with high self-assessment reported a high increase in confidence. This could suggest that students who already perceived themselves as competent perceived the training as confirmation of their abilities. Students with low self-assessment, on the other hand, may have related the training more strongly to their own perceived insecurities, potentially resulting in a smaller gain in confidence. Similar correlations have been observed in a quasi-experimental study in Nigerian Colleges of Education. Aikbekaen and coworkers examined the moderating effect of academic self-efficacy on educational interventions, such as Goal-Setting Therapy and Study Skills Training, and found that students with higher self-efficacy showed greater improvements in academic motivation and performance [39].

Before PT-BET, students rarely engaged in small groups to prepare for exams (Fig. 5a). Their willingness to do so increased over time (Figs. 5c and 6). The majority of students stated that they wanted to use this form of learning more frequently in the future. This may indicate that although many students were aware that cooperative learning methods could be helpful, they lacked distinct structures or positive experiences to implement them on their own initiative. PT-BET may have provided an important impulse here and may have made a practical model accessible. The uptake of peer learning has been shown to depend strongly on the availability of structured and guided formats [40]. These may include scheduled sessions and moderation, that could lead to positive outcomes, while less structured peer learning activities might be primarily used by already active students.

Furthermore, studies suggest that small groups can be particularly effective when learners have different levels of prior knowledge. However, similar benefits can also occur in groups with comparable levels of knowledge [41].

The longitudinal comparison between T1 and T2 suggests that repeated exposure to the PT-BET format may be associated with stronger improvements in stress management, self-assessment, and trust strengthening (Fig. 8a, b,c). This suggests that repeated training may be more beneficial than a single session for consolidating adaptive stress regulation and self-assessment. This is consistent with the concept of deliberate practice, which highlights repeated, structured training that includes feedback as a key mechanism for improving performance [42]. In contrast, peer group preparation and students’ intention to prepare for exams in peer groups remained largely unchanged between T1 and T2 (Fig. 8d, e). Responses were balanced, with most students reporting no meaningful change over time, suggesting a gap between perceived benefits and behavioral implementation that should be addressed in more detail for future optimization of the intervention.

The assessments by the tutors, who recorded exam-relevant communication skills, also suggested improvements in the categories of structure, response density, and expression (Fig. 9). These observations may be related to the switch between the roles of examiner, examinee and observer. When learners with different levels of prior knowledge interact, they may need to repeatedly rephrase complex concepts in simpler terms and explain them, which may help to consolidate understanding and identify gaps in their own knowledge. By actively examining topics, students not only had to reproduce content, but also had to be able to structure and reflect on it. The observer role may have helped students recognize important aspects of effective communication and internalize these strategies. Similar findings had been observed in other studies, which mainly relate to doctor-patient interactions, but also show that actively taking on different roles could improve one’s own competence [43–45].

Even though no measurable effects on grades were yet apparent in the midterm exam (Fig. 10a), positive trends were observed in M1 after the fourth semester (Fig. 10b). The grade distribution in 2025 showed more very good and good results, while very poor grades were less common than in previous years. However, these observations have to be considered in the context of various confounding factors, such as different examination boards. In addition, compared to the pandemic cohorts, distance learning was no longer in place and the sense of belonging among students may have been stronger again. Institutional monitoring data from the University of Duisburg-Essen indicated a significant improvement in the social integration of students after the COVID-19 cohorts and support this assumption [46]. Despite these influencing factors, numerous studies may potentially support the hypothesis that PT may be associated not only with subjectively perceived advantages but also with objectively measurable improvements: for example, higher exam grades in a biology course [15] and better performance in practical examinations (OSCE) of clinical skills [47]. Thus, PT-BET may represent a promising concept for targeted preparation of OE. Students may benefit in particular from a reduction in their subjectively perceived stress levels, increased confidence in their own exam abilities, improved exam rhetoric, and increased use of peer groups as a learning strategy.

### Strengths and limitations

This study highlights that the practical implementation of PT-BET in real, exam-like situations is feasible. In addition, both subjective data and objective assessments were collected, which increased the validity of the results. Repeated measurements over several semesters allowed changes over time to be tracked. The standardized PT-BET and feedback structure also contributed to the reliability of the results and their transferability to other student groups.

Despite the promising results, several limitations have to be considered. As the study did not include a control group within the same semester, this limits the ability to draw causal conclusions from the observed results. By its very nature, this design is susceptible to threats to internal validity, particularly maturation effects and regression to the mean. Therefore, it cannot therefore be determined whether the observed improvements are attributable to the PT-BET intervention, the regular course of the semester, the effects of repeated exposure, increased familiarity with physiological content, cohort effects, or post-pandemic normalization processes. However, we did not consider it to be ethically justifiable to deprive students of the potential benefits of PT-BET. The measurement instruments were recorded subjectively, and the Likert scales (1–5 or 1–6) used only allowed for limited differentiation of subtle differences. Variations among tutors and group dynamics may have influenced performance assessments. However, this heterogeneity reflects authentic teaching conditions. The persistence of the observable effects despite this inherent variance therefore indicates a certain robustness and generalizability of the intervention. The distribution of grades in the midterm exam and in the oral part of the M1 may also have been influenced by external factors such as varying exam requirements by different lecturers or variation between cohorts. In this context, comparisons of exams and grades across different M1 cohorts (2018, 2021, 2024, 2025) should be interpreted with caution, as these cohorts are not directly comparable. Potential confounding factors include differences in examiners, changes in the difficulty level of exams, cohort-specific performance differences, pandemic-related disruptions, and curriculum changes. Consequently, these comparisons should be viewed as exploratory and descriptive and should not be interpreted as evidence of causal relationships. Importantly, the M1 examination outcomes represent cohort-level data including both participants and non-participants of the PT-BET intervention. Finally, no demographic characteristics were recorded, and the results refer exclusively to the students’ cohort of Essen.

### Perspectives

Future studies could examine the extent to which the effects of PT-BET persist in the long term and how they affect different groups of students. Since PT-BET has only been used in the preclinical section of medical studies so far, future research could examine the extent to which the format is also effective in later stages of study, for example with the background to effectively practice physician-patient communication. It would also be valuable to compare the format with alternative teaching methods, such as exercises led by faculty members or lecture-based and exercise-oriented formats. In addition, it could be investigated which components are particularly decisive for reducing stress and building trust in order to optimize training programs in a targeted way.

### Practical implications for medical education

The results of this study show that structured peer teaching formats such as PT-BET can be a low threshold yet effective way of preparing students for oral exams while promoting self-efficacy expectations and communication competencies.

For successful implementation, it seems crucial that.

- peer teaching is not left to informal self-organization, but is integrated into a clearly structured and curriculum-embedded didactic framework.
- exam scenarios are realistic.
- the roles of students are clearly defined as either teacher or learner.
- tutors are trained effectively in observing the students’ performance as teachers/learners and in giving standardized, appreciative feedback.

PT-BET may then be applicable to support cognitive elaboration processes and to promote metacognitive reflection on one´s own learning and response process. Trained tutors play a central role in this, as a moderated learning environment strengthens both the quality of the feedback and psychological safety within the group. Peer teaching should be understood as preparatory, didactically structured learning support and not as a substitute for authentic exam situations.

## Conclusion

PT-BET shows promise as a feasible and potentially effective approach for preparing (medical) students specifically for OE situations. The supervised exchange on an equal footing strengthened both the students’ exam rhetoric and self-confidence, while at the same time it reduced their stress levels. It therefore seems sensible to integrate such trainings into preclinical education at an early stage in order to introduce students to exam-relevant communication situations right from the start. However, as the study is descriptive and hypothesis-generating in nature, further controlled prospective studies are needed to confirm these findings.

## Data Availability

The datasets used and/or analysed during the current study are available from the corresponding author on reasonable request.

## Abbreviations

ANOVA: Analysis of variance
BGBI.: Bundesgesetzblatt (Federal Law Gazette, Germany)
COVID: 19-coronavirus disease 2019
M1: first section of the German National Medical Licensing Examination
M3: third section of the German National Medical Licensing Examination
MTE: Midterm Exam
OE: Oral examination
OSCE: Objective structured clinical examination
PT: Peer teaching
PT: BET-peer teaching-based exam training
STEM: Science, technology, engineering and mathematics
T1: training round 1
T2: training round 2
UME: University Medicine Essen
