# Correction: M^1^ündlich – S(t)imulation of competency-oriented learning in physiology

**DOI:** 10.1186/s12909-026-10291-x

**Published:** 2026-09-02

**Authors:** Lisa Eiring, Sven Benson, Joachim Fandrey, Katrin Prost-Fingerle, Sandra Winning

**Affiliations:** 1 Institute for Physiology, University of Duisburg-Essen, University Hospital Essen, Hufelandstraße 55, Essen, 45147 Germany; 2 Institute for Medical Education, Center of Translational Neuro- and Behavioral Sciences (C-TNBS), University of Duisburg-Essen, Essen, Germany

**Correction: BMC Medical Education 26, 957 (2026)**

**https://doi.org/10.1186/s12909-026-09661-2**

Following publication of the original article [1], authors would like to replace " In 2024 (*n* = 59), to In 2024 (*n* = 152), under the Grade distribution in the M1 oral examination section and update Fig. 10.

**Figure:**
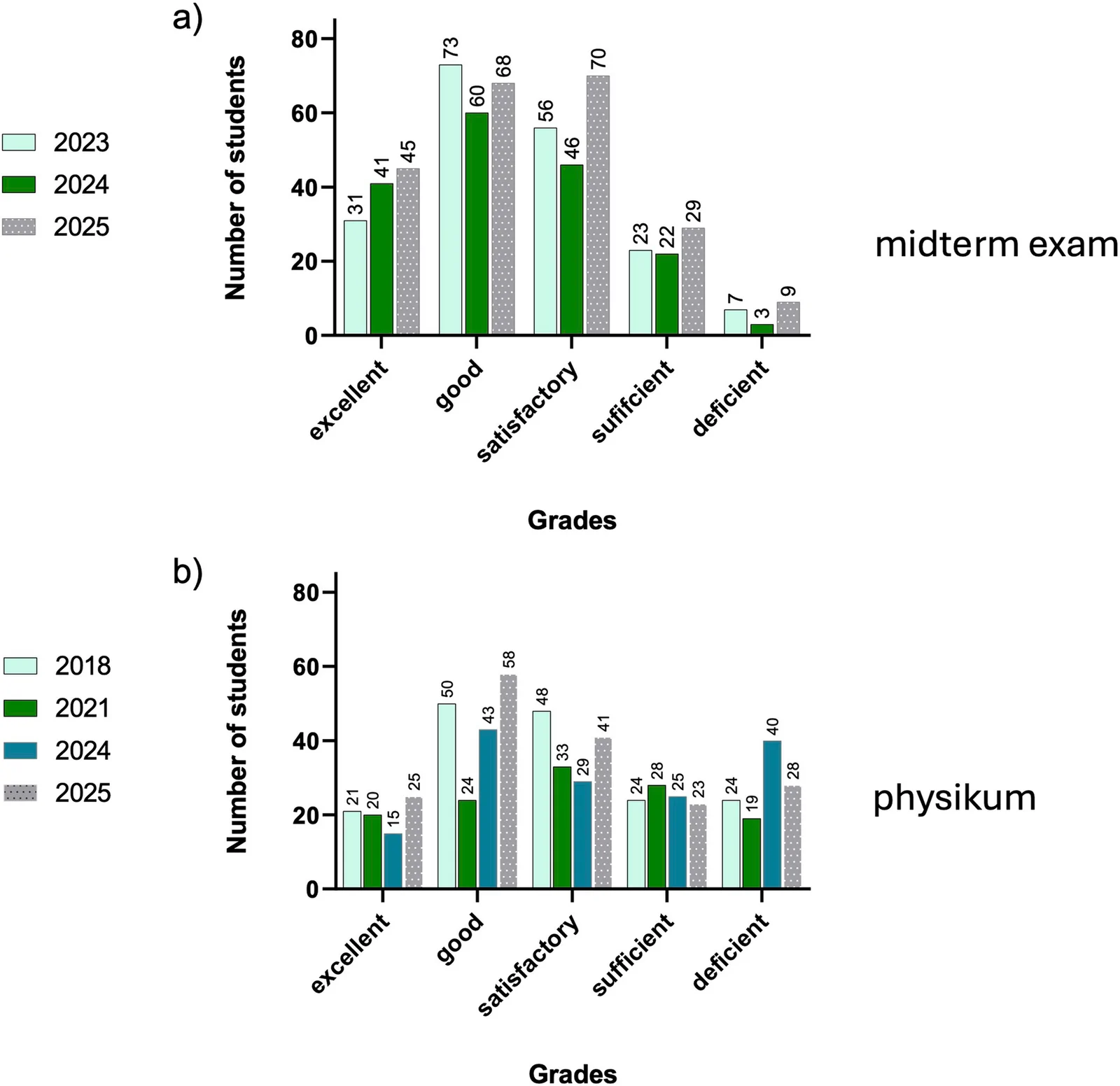

The original article [1] has been corrected.
